# Fracture resistance of roots filled with three different obturation techniques

**DOI:** 10.4317/medoral.17518

**Published:** 2011-12-06

**Authors:** Hüseyin S. Topçuoğlu, Hakan Arslan, Ali Keleş, Mustafa Köseoğlu

**Affiliations:** 1 DDS, research assistant. Department of Endodontics, Faculty of Dentistry, Atatürk University, Erzurum, Turkey; 2 DDS, PhD, assistant professor. Department of Endodontics, Faculty of Dentistry, İnönü University, Malatya, Turkey; 3 DDS, PhD, professor. Department of Endodontics, Faculty of Dentistry, Atatürk University, Erzurum, Turkey

## Abstract

Objectives: The aim of this study was to compare in vitro root fracture resistance following root canal filling with AH 26 using lateral condensation, BeeFill, and Thermafil techniques. Study Design: Eighty extracted human mandibular premolars with similar dimensions were selected. In order to standardize the roots, measurements were taken in two separate regions of the teeth—at the cemento-enamel junction and 8 mm apically from the junction—buccolingual as well as mesiodistal for every tooth. Teeth were then randomly divided into five groups (n=16). With the exception of the non-prepared group (Group 1), instrumentation was done in all groups. In group 2, instrumentation but no filling was performed; in group 3, the obturation was done with AH 26 + gutta-percha; in group 4, with AH 26 + BeeFill and in group 5, AH 26 + a Thermafil obturator was used. All the roots were mounted vertically in copper rings and filled with acrylic resin, exposing 8 mm of the coronal part. A universal testing machine was used for the strength test. Results: The results were analyzed using the one-way ANOVA test. The significance between the groups was tested with Temhane’s T2 test. The results indicate that instrumentation of root canals had a significant effect on fracture resistance (p<0.05). In addition, there were no differences between the root canal obturation techniques; furthermore, these techniques did not create a statistically important resistance to vertical fracture (p>0.05). Conclusions: The results suggest that instrumentation of root canals significantly weakens the tooth structure to fracture and the root canal obturation techniques that are used are not able to form reinforcement.

** Key words:**AH 26, obturation technique, vertical fracture.

## Introduction

An important cause of endodontic failure is root fracture, which is a serious clinical concern and results almost inevitably in extraction of the tooth or resection of the affected root (1-3). Endodontically treated teeth are dramatically susceptible to root fracture (4,5). It has been reported that vertical root fractures result largely from operative procedures performed in the root canal after the root canal treatment (6). Excessive loss of tissue during chemomechanical preparation (1,7,8) and excessive pressure during filling procedures (9) may play an important role in decreasing the resistance of teeth to fracture. The researchers have examined the effects of root canal preparation techniques (1), irrigation solutions (7), root canal filling materials (2,5,7,10-12) and root canal filling methods (4,7,12,13) on the tooth’s resistance to fracture.

Clinicians have long sought to reinforce the remaining tooth structure. Coronal reinforcement has been suggested with adhesive dental materials (7,14), crown placements (11,15), or fiber posts (15,16) to prevent unfavorable fractures after obturation. However, in some cases, even properly restored teeth may fracture. Therefore, one of the goals of filling the root canal must be to reinforce the root canal dentin to increase fracture resistance (17).

It is suggested that materials that can adhere to the root canal dentin surface will strengthen the remaining tooth structure (7,10). It was noted that AH 26 has a very good adhesion capability to dentin, as well as to gutta-percha (18-20). It has also been found that this effective adhesiveness capability does not change with heat application (21,22) , and that when used with gutta-percha, it increases resistance to vertical fracture (17,21).

Cold lateral condensation, warm vertical condensation (BeeFill), and Thermafil techniques are canal-filling methods widely used in endodontics; they are very different from each other when it comes to implementation. It is known that the different implementation procedures weaken the roots in different ways (12). At the same time, the possible increase in resistance to vertical root fracture through mechanisms such as adaptation and mechanical locking (11,17) shows a difference in canal-filling goals as a result of these different procedures. The aim of the present study is to compare the fracture resistance of root canals filled with AH 26 and the cold lateral condensation, BeeFill, and Thermafil techniques.

## Material and Methods

Eighty extracted human mandibular premolars with single canals that were approximately of the same dimension were selected and stored in saline solution until required. In order to standardize the roots used, measurements were made from every specimen at the cementoenamel junction (CEJ) and 8 mm more apical from the junction, in the buccolingual as well as mesiodistal direction of every specimen. In this way, four measurements were taken for each specimen. The measurements were made using a digital compass (Guanglu, China). The following example measurements were included in the study the CEJ buccolingual diameter was 6.5 ± 0.3 mm and the mesiodistal diameter was 4.7 ± 0.2; in the 8 mm apical region, the buccolingual diameter was 4.8 ± 0.3 mm and the mesiodistal diameter was 3.1 ± 0.3. All teeth were examined with a microscope of 25× magnification to detect any preexisting fractures; only intact teeth were included. Teeth were sectioned from the CEJ with a diamond bur used at high speed. The verification of the samples being single canalled was done by an expert using a #15 canal file (Dentsply Maillefer, Ballaigues, Switzerland). Later during the instrumentation, only two samples were discovered to be double canalled and were removed from the experiment; these were replaced with two different samples of the same dimensions. Except in the non-prepared group (group 1), the working length was determined to be 1 mm short of the apical foramen using a size 15 K-file. The root canals were instrumented to an ISO size 45 file at the apex and flared using a # 4 Gates-Glidden drill (Mani, Japan). During the instrumentation, irrigation with 1 ml of 2.5% sodium hypochlorite (Wizard, Rehber Chemistry, Istanbul, Turkey) was provided and a final rinse of 1 ml of 15% ethylenediaminetetraacetic acid (Wizard, Rehber Chemistry, Istanbul, Turkey) was used in order to remove the smear layer. Root canals were then flushed with saline solution and dried with paper points. Teeth were randomly divided into five groups of 16 teeth each.

Group 1: In this group, no root canal instrumentation was carried out. A cavity for temporary filling was drilled into the canal to 1 mm below the CEJ with a #4 Gates Glidden bur.

Group 2: In this group, root canals were not obturated and the group served as control. The canal opening was sealed with Cavit (3M ESPE AG, Germany).

Group 3: The teeth were obturated with lateral condensation using AH 26 (Dentsply De Trey GmBH, Germany) and gutta-percha (Aceone-Endo, Aceonedent. Co. Geonggi-Do, Korea). AH 26 was mixed according to the manufacturer’s instructions and placed into the root canal with a lentulo spiral filler (Dentsply Maillefer, Ballaigues, Switzerland). A #45 master gutta-percha cone was fit to the working length. Then, the gap for accessory cones was created consecutively using the numbers 35, 30, 25, 20, and 15 finger spreaders (Dentsply Maillefer, Ballaigues, Switzerland). Excess gutta-percha was removed 1 mm below the canal opening. The canal opening was sealed with Cavit.

Group 4: The teeth were filled using the BeeFill (VDW, Munich, Germany). AH 26 was applied to the canal walls with a lentulo spiral filler. Number 45 master gutta-percha was fitted 3 mm short of the working length with a tug-back. The BeeFill down-packing device was used for obturation of the apical part of the root canal system. The coronal part of the root canal was filled with a backfilling device. The heated gutta-percha was vertically compacted with pluggers (Dentsply Maillefer, Ballaigues, Switzerland). The canal opening was sealed with Cavit.

Group 5: The prepared teeth were obturated using the Thermafil technique with a plastic carrier. AH 26 was placed into the root canal with a lentulo spiral filler. A size 45 Thermafil obturator with plastic carrier was heated in the Thermaprep® Plus Oven (Densply, Maillefer, Ballaigues, Switzerland). The heated obturator was slowly inserted into the canal to the previously determined working length. A plugger was used to condense the coronal gutta-percha around the carrier until the gutta-percha hardened. The canal opening was sealed with Cavit. All roots were stored in 100% humidity for two weeks to allow the sealer to set.

Preparation of the mechanical test

All the roots were mounted vertically in Copper rings (20 mm high and 20 mm in diameter), filled with acrylic resin (Imicryl, Konya, Turkey), exposing 8 mm of the coronal part. A universal testing machine (Instron Corp. MA, USA) was used for the strength test. The acrylic blocks were placed on the lower plate of the machine. The upper plate included a steel spherical tip with a diameter of 4 mm. The tip contacted a slowly increasing vertical force (1mm min-1) until fracture occurred. The force when the fracture occurred was recorded as Newtons. The results were analyzed using the one way ANOVA test (SPSS 10.0, SPSS, Chicago, USA). Significance between the groups was then tested with the Temhane’s T2 test. All statistical analysis was performed at the 95% level of confidence.

## Results

The distribution of median fracture values of each group are shown in ( Table 1). Temhane’s T2 test results indicated that preparation of the root canals significantly weakened the root structure to fracture (p<0.05). In addition, based on the results of this experimental study, there were no differences found between the root canal obturation techniques that were tested and these techniques did not create a statistically important resistance to vertical fracture.

**Table 1:**
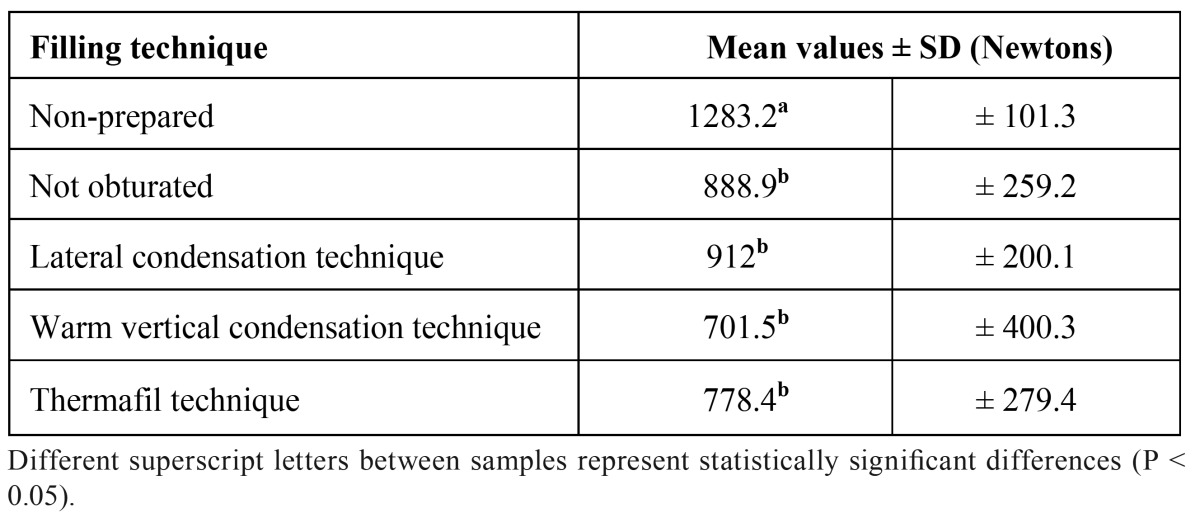
Force that is required to cause vertical root fracture (measured in Newtons).

## Discussion

There is a perception that root canal treatment weakens tooth structure and predisposes teeth to fracture (13). Researchers have examined the effects of different endodontic instrumentation and obturation techniques on vertical fractures, as well as how different root canal filling materials and techniques affect the resistance of the remaining tooth structure to vertical fractures (10,12,13,17,23). By standardizing the specimens, researchers have obtained valuable data showing that their experimental situations were appropriate. However, effective factors such as preserving situations of the teeth, the height of the part of the tooth that remains outside of the acrylic base, the angle at which the tooth is placed inside the acrylic, and the length and shape of the steel tip showed quite a bit of variance among the studies. Even though every study resulted in valuable information according to the requirements of the study, researchers could reach different conclusions based on such factors (11,17).

In this study, all of the controllable factors apart from the filling technique were standardized as much as possible. All roots were instrumented using the same technique. The age of the patient, the gutta-percha type used, and dentine sclerosis were not taken into account. In order to standardize the conditions, the study included two separate regions of the teeth—at the CEJ and 8 mm more apically from the junction—with similar buccolingual as well as mesiodistal dimensions, on single canal mandibular premolar teeth. It was mentioned that in previous studies measurements were done only the CEJ of the roots (5,7). In the present study, during the selection and standardization of the specimens, there were many roots with a similar diameter in the CEJ but that showed a difference in the 8 mm more apical section; because of this, they were not be included in the study. Thus, we selected roots that were as similar as possible and assigned them randomly into groups. Because of its good adhesion capability to dentin and gutta-percha, AH 26 was used in this study (18-20,24), and it has also been determined that this adhesion capability did not change with heat application (21,22). In this way, same adhesive capability was used for all three groups.

In this study, it was found that non-prepared group (Group 1) had higher resistance to vertical fracture than the prepared but unfilled group (Group 2) (p<0.05). The finding that preparation weakens roots and makes teeth susceptible to vertical fracture is supported by previous studies (4,5).

This study showed that there was not statistical difference between the canal filled groups and the unfilled group (p> 0.05). It has been reported that AH 26 was better than other tested materials and showed a resistance to fracture compared to unfilled group (17). It has also been found that there was no difference between the materials that were compared to AH 26 and that it had more resistance compared to the unfilled group (5,13); however, it has also been shown that AH 26 provided less resistance than the materials that’s compared to and there was no difference with the unfilled group (12).

Lateral and vertical gutta-percha compactions and thermo-plasticized gutta-percha techniques involve different procedures in endodontic therapy. Researchers have examined these techniques in terms of issues such as sealing ability (25-27), caused to root strains (23), and the effect of resistance to vertical fracture (12). In this study, which evaluated the effects of filling techniques that include different procedures on vertical fracture resistance, it was found that there is no statistically significant difference between the filling techniques, and that these techniques cannot form a resistance to vertical fracture (p>0.05). In studies that examine the resistance of various sealers to vertical fractures, researchers have reported different results concerning lateral condensation techniques. Some studies indicated that the lateral condensation group had higher resistance to vertical fracture than the unfilled group (5,13,17), but there were also studies that indicate that there was no statistical difference between these techniques (7,11,12). In Teixeira’s study (12), which tested the effects of obturation and lateral condensation techniques on the fracture resistance, no statistically significant difference between these groups was found in relation to these two techniques and the unfilled group. The results that we attained from our research support this study. However, to our knowledge, there are no studies regarding the effects of Thermafil technique on fracture resistance.

The results of this research showed that advantages or disadvantages of filling techniques such as homogenous, voids, spreader tracts, lack of surface, adaptation and amount of sealer were not effective on reinforcement of the roots.

Under the conditions of this study, the resistance of the root to vertical fracture is decreased with instrumentation, and the root canal obturation techniques used are not able to provide reinforcement.
